# Fine‐tuned interactions between globular and disordered regions of single‐stranded DNA binding (SSB) protein are required for dynamic condensation under physiological conditions

**DOI:** 10.1002/pro.70109

**Published:** 2025-03-27

**Authors:** Zoltán J. Kovács, Péter Ecsédi, Gábor M. Harami, János Pálinkás, Mina Botros, Lamiya Mahmudova, Viktoria Katran, Dávid Érfalvy, Miklós Cervenak, László Smeller, Mihály Kovács

**Affiliations:** ^1^ ELTE‐MTA “Momentum” Motor Enzymology Research Group, Department of Biochemistry Eötvös Loránd University Budapest Hungary; ^2^ HUN‐REN–ELTE Motor Pharmacology Research Group, Department of Biochemistry Eötvös Loránd University Budapest Hungary; ^3^ Department of Biophysics and Radiation Biology Semmelweis University Budapest Hungary

**Keywords:** genome maintenance, liquid–liquid phase separation (LLPS), protein–protein interactions, single‐stranded DNA binding protein (SSB)

## Abstract

Increasing evidence points to the importance of liquid–liquid phase separation (LLPS)‐driven protein condensation in both eukaryotic and bacterial cell physiology. The formation of condensates may involve interactions between both structured (globular) domains and intrinsically disordered protein regions and requires multivalency that is often brought about by oligomerization. Here we dissect such contributions by assessing engineered variants of bacterial (*Escherichia coli*) single‐stranded DNA binding (SSB) protein whose condensation has recently been implicated in bacterial genome metabolism. A truncated SSB variant (SSBdC, lacking the conserved C‐terminal peptide (CTP)) was used to assess the importance of interactions between SSB's globular oligonucleotide/oligosaccharide binding (OB) domain and the CTP. We show that OB–CTP interactions are essential for dynamic condensation in physiological (crowded, glutamate‐rich) environments. Via assessment of a protein variant (SSB^H55Y^) from the known thermosensitive *ssb‐1* mutant, we also show that the perturbation of OB–OB contacts significantly impairs the stability of SSB tetramers and results in thermally induced protein aggregation, underscoring the importance of multivalence brought about by stereospecific contacts. Our data point to adaptive fine‐tuning of SSB interactions to physiological condensation and demonstrate that SSB represents a versatile system for selective engineering of condensation‐driving interactions between globular and disordered regions.

## 1 INTRODUCTION

Single‐stranded (ss) DNA segments are continuously generated during various processes of genome metabolism, including DNA replication, recombination, and repair. All living cells express single‐stranded DNA‐binding (SSB) proteins that protect ssDNA from nucleolytic degradation and prevent ectopic DNA secondary structure formation (Antony & Lohman, 2019; Ashton et al., 2013; Croft et al., 2019; Shereda et al., 2008). Besides this function, SSBs are network hubs physically interacting with numerous genome maintenance proteins, thereby coordinating their activities and localization (Bianco, 2021).

The “prototypic” bacterial SSB from *E. coli* forms a homotetramer of 19‐kDa subunits, each of which consists of an N‐terminal oligonucleotide/oligosaccharide‐binding (OB) domain (amino acid residues (aa) 1–112) and an intrinsically disordered linker (IDL, aa 113–177) containing a highly conserved C‐terminal peptide (CTP) segment (aa 169–177) (Kozlov et al., 2015; Raghunathan et al., 2000). The OB domain is responsible for tetramerization and ssDNA binding (Bujalowski & Lohman, 1991a; Kozlov et al., 2017; Raghunathan et al., 1997, 2000). Although the precise mechanistic role of the IDL has been elusive, cells expressing IDL‐truncated or ‐modified (but CTP‐containing) SSB variants showed increased sensitivity to UV radiation, highlighting the significance of the IDL in DNA repair (Bonde et al., 2023; Kozlov et al., 2015). The CTP (aa sequence MDFDDDIPF in *E. coli*) exhibits high conservation among all Bacteria (Bonde et al., 2023), with a primary function of establishing stereospecific interactions with numerous protein partners including nucleases, glycosylases, polymerases, and helicases (Antony & Lohman, 2019; Mills et al., 2017; Shereda et al., 2008). Moreover, the CTP competes with ssDNA for binding to SSB's OB domain, leading to intertetramer OB–CTP interactions (Kozlov et al., 2015; Marceau et al., 2011).

Dynamically regulated condensation of proteins and nucleoprotein complexes into membraneless organelles, brought about by liquid–liquid phase separation (LLPS), is being recognized as a central organizing principle in an increasing number of cell physiological processes (Yoshizawa et al., 2020). LLPS processes are primarily driven by weak secondary interactions between molecules including ionic, dipole–dipole, cation–π, and π–π interactions (Alberti et al., 2019). Several regions within proteins promote the formation of LLPS processes, including oligomerization domains, nucleic acid‐binding domains, prion‐like regions, repeating short linear motifs and their binding domains, as well as disordered regions (Gomes & Shorter, 2019; Mittag & Parker, 2018). Phase separation can happen at macromolecule concentrations where interactions between macromolecules are energetically more favorable than the entropy‐driven process that leads to the formation of the homogeneous phase. During the process, two phases are formed: a larger volume solution phase with a low macromolecule concentration, and a smaller volume condensed phase with a high macromolecule concentration. The phase‐separated state has the lowest Gibbs free energy, and the process occurs spontaneously (Banani et al., 2017; Brangwynne et al., 2015). These condensates can act as reaction compartments in which enzymes and substrates can accumulate, thus increasing reaction rates and specificity. Protein condensates are also ideal spaces to sequestrate components. Moreover, condensates can serve as stress sensors due to their rapid response ability (Alberti et al., 2019; Hyman et al., 2014; Pancsa et al., 2019).

Besides wide‐ranging examples in eukaryotic cells (Banani et al., 2017; Yoshizawa et al., 2020), the importance of intracellular condensation in bacteria is receiving increased attention (Azaldegui et al., 2021). We and others recently described dynamic, ssDNA‐regulated condensation properties for *E. coli* SSB, with implied roles in bacterial genome metabolism (Harami et al., 2020; Kozlov et al., 2022). However, the structural determinants and the underlying molecular mechanisms of SSB condensation are still imperfectly understood. Besides the contribution of multivalent, stereospecific OB–CTP interactions between SSB tetramers, the IDL has also been proposed to affect condensation via intertetramer IDL–IDL interactions and/or controlling CTP accessibility (Harami et al., 2020; Kozlov et al., 2022). Accordingly, SSB mutant variants in which the IDL was replaced either with a short Gly–Gly dipeptide segment or the IDL of condensation‐incompetent *Plasmodium falciparum* SSB showed no condensation (Kozlov et al., 2022).

Here we sought to dissect the contributions of stereospecific OB–OB and OB–CTP interactions from non‐stoichiometric ones between IDLs by characterizing the effect of (i) CTP deletion using a CTP‐less SSB variant (SSBdC, comprising aa 1–170), and (ii) perturbation of tetramerization‐mediating OB–OB interactions using a previously described SSB variant harboring the H55Y aa substitution (SSB^H55Y^, mutant termed *ssb‐1*) (Bujalowski & Lohman, 1991b; Meyer et al., 1980; Tessman & Peterson, 1982). We find that CTP deletion renders SSBdC condensation dependent on molecular crowding, while it precludes condensation in physiological, glutamate‐rich environments. Nevertheless, the dynamic regulation of SSB condensation by ssDNA is retained by SSBdC under permissive conditions, showing that the competition between ssDNA and the CTP for binding to the OB fold is not the sole determinant of this phenomenon. Moreover, SSB's interaction partner, RecQ DNA helicase, as well as its variants (R425A, R499A) that are perturbed with regard to their SSB interaction, show selective enrichment in both SSB and SSBdC condensates, suggesting that CTP‐partner interactions are not essential for partner recruitment into SSB condensates. We find significantly reduced thermal stability and signs of thermally induced aggregation for SSB^H55Y^ tetramers, in contrast to wild‐type SSB, identifying molecular determinants of *ssb‐1* phenotypes. Taken together, our data reveal significant contributions for both OB–CTP and OB–OB interactions to SSB's condensation mechanism, and highlight that SSB represents a versatile system for engineering interactions that together bring about dynamically regulated protein condensation.

## 2 RESULTS

### 2.1 Crowding‐dependent SSB condensation in the absence of the conserved CTP

**FIGURE 1:**
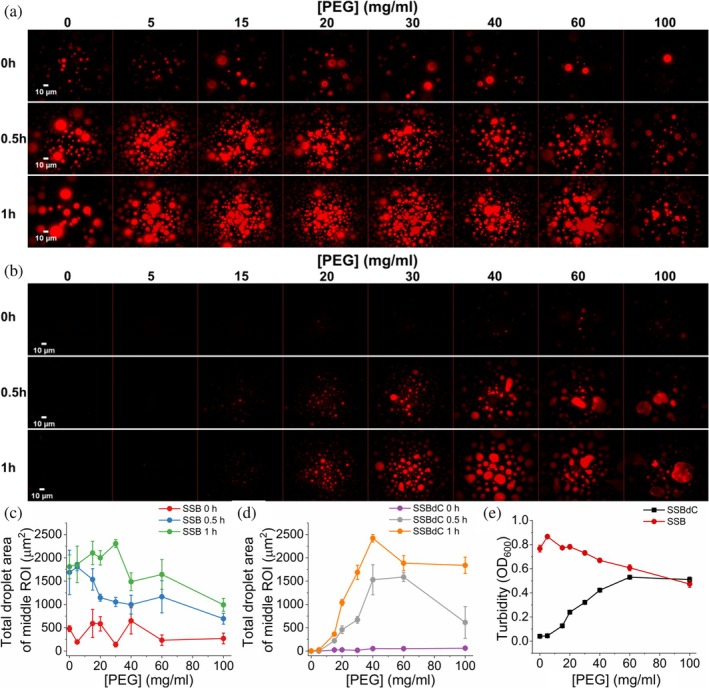
SSB condensate formation becomes crowder‐dependent in the absence of the conserved C‐terminal peptide (CTP). (a), (b) Epifluorescence microscopic images obtained upon mixing 15 μM SSB (protein concentration optimized based on Harami et al., 2020) and 0.15 μM Alexa555‐labeled SSB; (a) wild‐type (WT) SSB, hereinafter referred to as SSB; (b) SSBdC construct comprising amino acid residues (aa) 1–170, lacking the CTP in standard LLPS buffer complemented with polyethylene glycol (PEG 20,000, hereinafter referred to as PEG) at the indicated concentrations. Samples were incubated for the indicated times before imaging. (c), (d) PEG concentration dependence of the total droplet area of (c) SSB and (d) SSBdC fluorescence images from panels (a), (b), determined from the middle ROI (region of interest, see Methods). Means ± SEM are shown for *n* = 3. (e) PEG concentration dependence of turbidity (*OD*_600_) values recorded upon mixing 15 μM unlabeled SSB or SSBdC in standard LLPS buffer with indicated concentrations of PEG, followed by 1‐min incubation. Means ± SEM are shown for *n* = 3. Error bars are within symbols when not visible.

Previously we found that, unlike SSB, SSBdC required the presence of bovine serum albumin (BSA) as a crowding agent for condensate formation (Harami et al., 2020; see Figure S1 for electrophoretic assessment of protein preps used in the current study). Similarly to BSA, molecular crowder polyethylene glycol (PEG 20,000, hereinafter referred to as PEG) is also capable of initiating SSBdC condensation in a concentration‐dependent manner, with half‐maximal effect at around 30 mg/mL PEG. However, at higher concentrations, PEG had a partial inhibition on SSBdC condensation (Figure 1a–d). Note that slight inhibition was also seen previously in the case of SSB condensation when BSA was used at 150 mg/mL concentration (Harami et al., 2020). The observations of turbidity (*OD*_600_) measurements were in line with the microscopic results (Figure 1e). The observed inhibition could be due to intensive condensation caused by the increasing surface tension of the crowder. When droplet formation is extreme, condensates can easily collide to form continuous LLPS layers. These layers do not appear during microscopic analysis and scatter light less efficiently, leading to decreased turbidity signals. Based on these and previous results (Harami et al., 2020), in our subsequent experiments, we chose to use 30 mg/mL PEG and 150 mg/mL BSA concentrations, at which both SSB and SSBdC showed significant extents of condensation.

### 2.2 OB–CTP interactions are not essential for ssDNA inhibition of condensation

**FIGURE 2:**
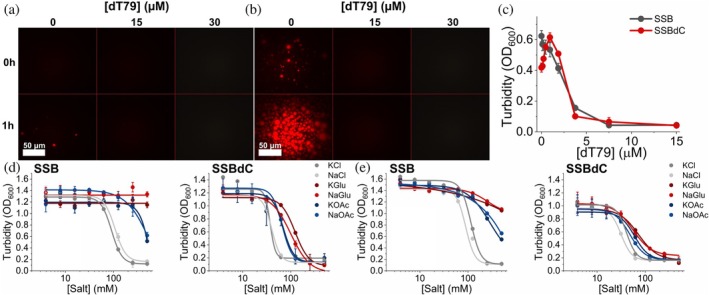
ssDNA inhibits condensate formation by both SSB and SSBdC under permissive (crowded, glutamate‐free) conditions; SSB condensation becomes glutamate‐inhibited in the absence of the CTP. (a), (b) Epifluorescence microscopic images obtained upon mixing 30 μM SSBdC (protein concentration optimized based on Harami et al. (2020)) and 0.3 μM Alexa555‐labeled SSBdC in standard LLPS buffer with indicated concentrations of 79‐mer homo‐deoxythymidine (dT_79_) ssDNA, in the (a) absence and (b) presence of 150 mg/mL BSA as molecular crowder. Images were captured immediately or after 1‐h incubation as indicated and were not background corrected. (c) dT_79_ concentration dependence of turbidity (*OD*_600_) values recorded upon mixing 15 μM unlabeled SSB and SSBdC and the indicated dT_79_ concentrations in standard LLPS buffer containing 30 mg/mL PEG, followed by 1‐min incubation. Means ± SEM are shown for *n* = 3. (d), (e) Turbidity (*OD*_600_) values recorded upon mixing 10 μM unlabeled SSB or SSBdC in standard LLPS buffer containing (d) 150 mg/mL BSA or (e) 30 mg/mL PEG and the indicated concentrations of different salts, followed by 1‐min incubation. Means ± SD are shown for *n* = 3. Solid lines show best‐fits based on the Hill equation. See Table S1 for half‐maximal effective concentration values.

Previously we showed that ssDNA hinders SSB condensation, proposedly by competing with the CTP for binding sites on the OB domain (Harami et al., 2020). In epifluorescence microscopic experiments using fluorescently labeled SSBdC, we found that the BSA‐assisted condensation of SSBdC is also inhibited by the addition of 79‐mer homo‐deoxythymidine ssDNA oligonucleotide (dT_79_) (Figure 2a, b). For quantitative assessment of this effect, we conducted turbidity measurements in which SSB and SSBdC were titrated with increasing dT_79_ concentrations in the presence of PEG. Interestingly, unlike that for SSB, we found a moderate enhancement in SSBdC turbidity at low ssDNA concentrations (>10 SSBdC tetramers per dT_79_ molecule), suggesting that an ssDNA scaffolding effect even slightly facilitates SSBdC condensation under these conditions (Figure 2c). However, turbidity eventually decreased with increasing ssDNA concentrations, with SSBdC exhibiting a similar ssDNA saturation profile to that of SSB (Figure 2c). These data show that OB–CTP interactions are not essential for ssDNA regulation of SSB condensation in crowded, glutamate‐free conditions.

### 2.3 CTP deletion renders SSB condensation sensitive to glutamate, the main physiological anion in *E. coli* cells

Previously we showed that chloride ions exert a strong inhibitory effect on SSB condensation, while glutamate (Glu) was shown to be permissive (Harami et al., 2020) and to enhance the process (Kozlov et al., 2022). Here, we assessed the impact of Cl^−^, acetate, and Glu on SSB and SSBdC condensation in the presence of PEG and BSA to permit SSBdC condensation (see above). In line with previous findings (Harami et al., 2020), Glu did not elicit a decrease in SSB turbidity, while the strong condensation‐inhibiting effect of Cl^−^ seen earlier in the absence of crowders (Harami et al., 2020) was found to be unchanged in their presence. The effect of acetate was found to be of intermediate magnitude. Importantly, we found SSBdC condensation to be inhibited by all salts with similar concentration dependence. SSBdC condensation was markedly more salt‐sensitive than that of SSB in all cases (Figure 2d, e, Table S1). These results show that SSBdC is unable to undergo condensation under glutamate‐rich conditions in the presence of crowders, as pertinent to the environment within *E. coli* cells.

### 2.4 The SSB binding partner RecQ helicase becomes enriched in SSB condensates even in the absence of the RecQ–CTP interaction

**FIGURE 3:**
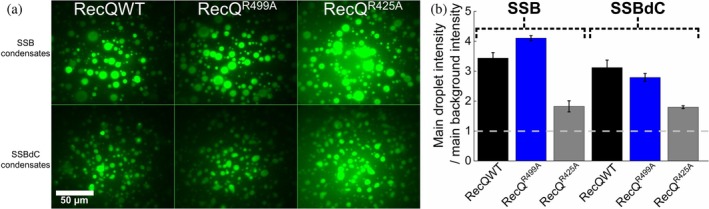
The SSB binding partner RecQ helicase becomes enriched in SSB condensates even in the absence of the SSB CTP, and with amino acid substitutions weakening RecQ's SSB interface. (a) Epifluorescence microscopic images obtained upon mixing 30 μM unlabeled SSB or SSBdC and 180 nM of Alexa488‐labeled WT RecQ helicase (RecQWT) or RecQ variants (protein concentration optimized based on Harami et al. (2020)) harboring the R499A (RecQ^R499A^) or R425A (RecQ^R425A^) aa substitutions in standard LLPS buffer containing 30 mg/mL PEG. Samples were incubated for 10 min before imaging. Images were not background corrected. (b) Enrichment of RecQ constructs in SSB and SSBdC condensates, determined from the ratio of the mean signal intensity within droplets and the mean background intensity. Background‐uncorrected fluorescence images were used for analysis. Means ± SEM are shown for *n* = 3 (see “Methods” for further details).

Previous studies have established SSB's role as a central interaction hub in bacterial genome metabolism, with the CTP being a key structural element that physically interacts with SSB's numerous binding partner proteins (Antony & Lohman, 2019). Earlier we showed that RecQ helicase, a key SSB interacting partner, becomes enriched inside SSB condensates (Harami et al., 2020). We detected enrichment even for the RecQ^R425A^ and RecQ^R499A^ variants harboring amino acid substitutions in the so‐called winged helix domain (WHD, RecQ's primary SSB interacting site (Harami et al., 2013; Harami et al., 2020; Shereda et al., 2009)). This finding was unexpected as these RecQ variants bind SSB with 50‐ and 300‐fold lowered affinities compared to RecQ, respectively (Harami et al., 2020). In the present study, we found that each RecQ construct became significantly enriched also in condensates formed by the SSBdC construct (Figure 3), despite the finding that they mainly interact with SSB through the CTP in dilute aqueous medium (Shereda et al., 2007). This finding suggests that the CTP‐WHD interaction is not the sole determinant of the interaction between the SSB and RecQ proteins, at least under circumstances that are pertinent within SSB condensates.

### 2.5 The presence of the CTP facilitates SSB condensation over an extended pH range

**FIGURE 4:**
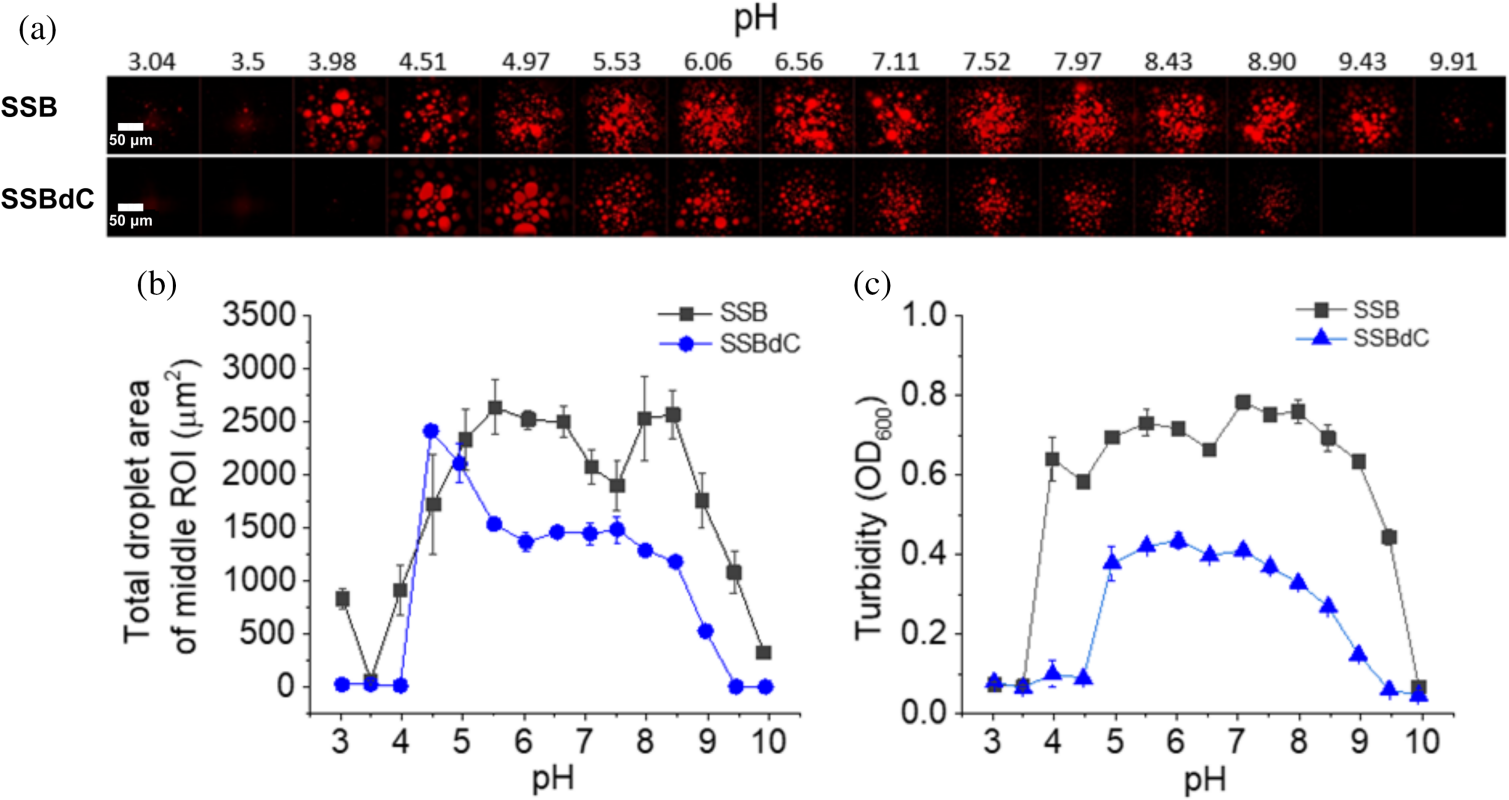
CTP deletion slightly narrows the pH range for SSB condensate formation. (a) Epifluorescence microscopic images obtained upon mixing 15 μM SSB and 0.15 μM Alexa555‐labeled SSB (upper row) or 15 μM SSBdC and 0.15 μM Alexa555‐labeled SSBdC (lower row) in standard LLPS buffer complemented with 30 mg/mL PEG, buffered to the indicated pH values. The buffering agent was citrate, MES, and Tris, in pH ranges 3–4, 4.5–6.5, and 7–10, respectively (see also “Methods”). Samples were incubated for 1 h before imaging. (b) pH dependence of total droplet area of SSB and SSBdC condensates determined from the middle ROI of epifluorescence images of panel (a). Means ± SEM are shown for *n* = 3. (c) pH dependence of turbidity (*OD*_600_) values recorded upon mixing 15 μM unlabeled SSB or SSBdC in conditions described for panel (a), followed by 1‐min incubation. Means ± SEM are shown for *n* = 3.

Since ionic interactions between proteins have been implicated as essential for protein condensation (Murthy et al., 2019), we investigated the process by using labeled SSB and SSBdC in the presence of PEG in epifluorescence microscopic experiments in a broad pH range (pH 3–10, in carefully buffered solutions, see Methods) (Figure 4a, b). SSB showed condensation over a wide pH range (4–10), while below pH 4 only limited amorphous aggregation occurred. SSBdC condensation was apparent in a narrower pH range between 5 and 9, presumably due to the lack of the stabilizing effect caused by the CTP. Results of turbidity measurements were in line with those of microscopic experiments (Figure 4c).

### 2.6 OB–CTP interactions are enhanced by PEG, slightly inhibited by glutamate, and strongly inhibited by ssDNA and chloride ions

**FIGURE 5:**
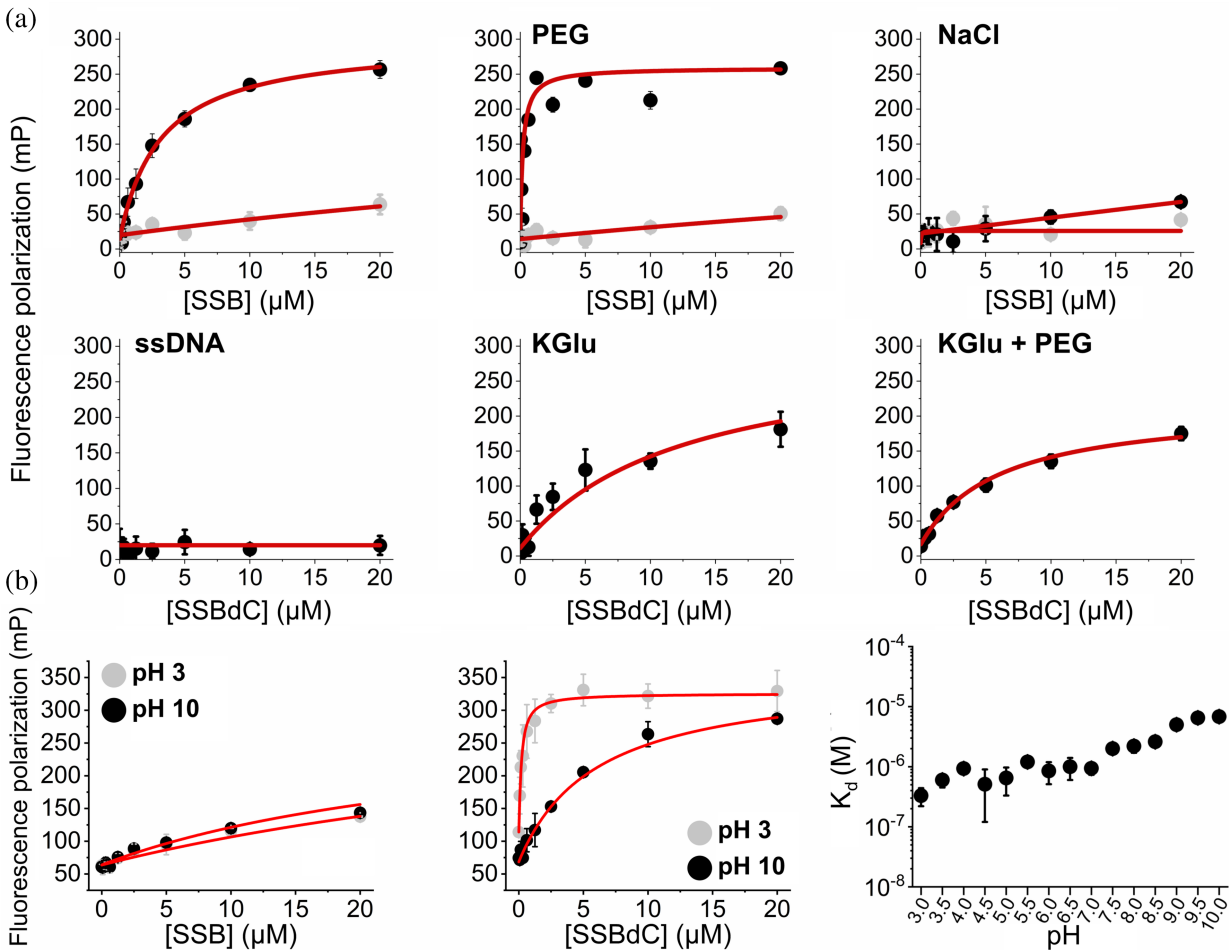
Binding of the isolated SSB C‐terminal peptide (CTP) to the SSBdC construct is enhanced by PEG, slightly inhibited by glutamate, and strongly inhibited by ssDNA and chloride ions; CTP binding to SSBdC is pH‐dependent. (a) Shown are fluorescence polarization titrations of 15 nM fluorescein‐labeled SSB C‐terminal peptide (flu‐CTP) with increasing concentrations of SSBs (SSB gray; SSBdC, black) in the absence of PEG (upper left panel); in the presence of 30 mg/mL PEG (upper middle panel); and in the presence of 300 mM NaCl (upper right panel). Note that the peptide binds weakly to the SSB compared to SSBdC; thus, the binding in the presence of ssDNA (20 μM dT_79_) (lower left panel), 300 mM KGlu (lower middle panel), or 300 mM KGlu and 30 mg/mL PEG (lower right panel) was carried out using SSBdC only. (b) Fluorescence polarization titrations of 15 nM flu‐CTP with increasing concentrations of SSB (left) or SSBdC (middle) at different pH values. Note that only the two extremes (pH 3, pH 10) are presented. The right panel shows the pH dependence of the determined *K*_d_ values of flu‐CTP binding to SSBdC. Data points are the means ± SD of three independent experiments. Solid red lines show best fits using a quadratic binding equation. Best‐fit *K*_d_ values are listed in Table S2.

As the above findings pointed to the role of charged interactions, potentially between the CTP and the OB domain, in SSB condensation, we also assessed the binding of fluorescently labeled isolated CTP (flu‐CTP) to SSB and SSBdC in fluorescence polarization titrations under various conditions (Figure 5, Table S2). As expected, the isolated CTP showed very weak binding to SSB, probably due to its inability to effectively compete with SSB's endogenous CTP for OB fold interactions, as the endogenous CTP is present at a high effective concentration due to spatial proximity to the OB domain. In contrast, flu‐CTP binding to SSBdC was apparent even in the absence of a crowding agent, and it was further enhanced by PEG, slightly inhibited by glutamate, and strongly blocked either by ssDNA or by chloride ions (Figure 5a, Table S2). Intriguingly, we found that the interaction of flu‐CTP with SSBdC showed a gradual weakening with increasing pH throughout the examined broad pH range (pH 3–10) (Figure 5b, Table S2).

### 2.7 Condensation propensity is retained by the SSB^H55Y^ variant harboring a perturbed OB–OB interface

**FIGURE 6:**
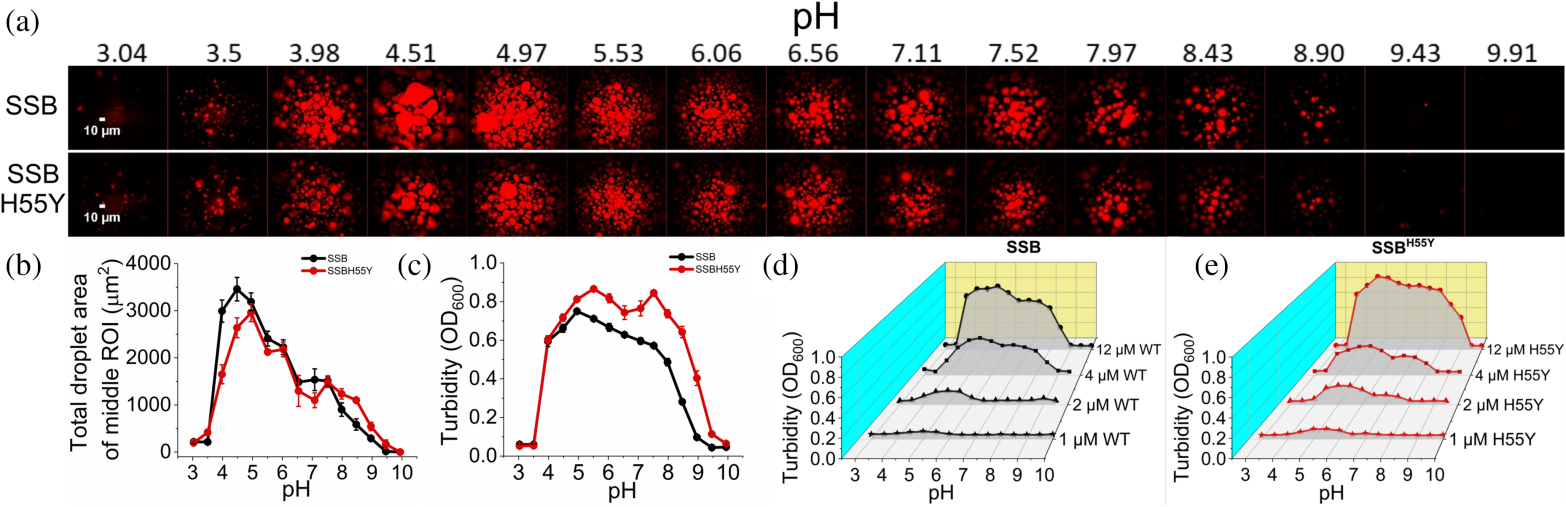
SSB^H55Y^ retains pH‐dependent condensation characteristic of wild‐type SSB. (a) Epifluorescence microscopic images obtained upon mixing 15 μM SSB and 0.15 μM Alexa555‐labeled SSBs (upper row: SSB, lower row: SSB^H55Y^) at different pH values in buffers described for Figure 4, with the difference that PEG was not used in the current experiments. Samples were incubated for 1 h before imaging. (b) pH dependence of total droplet area of SSB and SSB^H55Y^ condensates determined from the middle ROI of epifluorescence images of panels (a). Means ± SEM are shown for *n* = 3. (c) pH dependence of turbidity (*OD*_600_) values recorded upon mixing 15 μM unlabeled SSB or SSB^H55Y^ in conditions described for panel (a), followed by 1‐min incubation. Means ± SEM are shown for *n* = 3. (d), (e) pH dependence of turbidity (*OD*_600_) values recorded for (d) unlabeled SSB and (e) SSB^H55Y^ at the indicated concentrations in standard LLPS buffer as described for panel (a), after 1‐min incubation. Means ± SEM are shown for *n* = 3 (error bars are mostly within symbols).

The H55Y amino acid substitution within the OB domain, harbored by the long‐known *ssb‐1* mutant, has been shown to result in thermosensitivity, reduced protein structural stability, and attenuated SSB tetramerization (Meyer et al., 1980; Williams et al., 1984). To assess the role of OB–OB interactions and protein tetramerization in SSB condensation, we purified and analyzed the SSB^H55Y^ variant. The ssDNA binding capability of SSB^H55Y^ was retained, according to our fluorescence anisotropy titrations using fluorescein‐labeled 36‐mer ssDNA oligonucleotide (Figure S2). Next, we investigated the condensation propensity of SSB^H55Y^ via epifluorescence microscopic experiments performed in the pH range 3–10, applying fluorescently labeled SSB protein in the absence of PEG (Figure 6a–c). In these experiments, SSB^H55Y^ showed WT‐like condensation features, and turbidity measurements also indicated WT‐like protein concentration dependence for SSB^H55Y^ condensation in the assessed range (1–12 μM, Figure 6d, e).

### 2.8 H55Y substitution markedly decreases, while glutamate markedly increases SSB tetramer thermostability

As SSB‐WT and SSB^H55Y^ exhibited similar condensation behavior within the investigated pH and protein concentration range in our fluorescence microscopic and turbidity measurements (Figure 6), we compared the thermostability of SSB and SSB^H55Y^ tetramers. We utilized ThermoFluor‐based assays (see “Methods”) to discern alterations in the dissociation temperature of the SSB tetrameric structure at physiological pH (7.5) at which histidine side chains are predominantly deprotonated, and at acidic (pH 5) conditions in which histidines are predominantly protonated. First, we tested the sensitivity of the method by recording ThermoFluor fluorescence temperature profiles of 15 μM SSB and SSB^H55Y^ at physiological pH (7.5) in the absence and presence of 200 mM KCl (condensation‐permissive and condensation‐abolishing conditions, respectively) (Figure S3 and Table S3). Apparent melting temperatures (*T*_m_, i.e., those at peak *d*(RFU)/*dT* values) were markedly lower (by 11–14°C) for SSB^H55Y^ compared to SSB, reflecting reduced structural stability of the modified protein variant. Importantly, the fluorescence profiles were not markedly affected by the presence of KCl, indicating that ThermoFluor selectively reports structural transitions associated with SSB tetramer disassembly and is largely insensitive to protein condensation (Figure S3). Intriguingly, *T*_m_ values were slightly higher (by 2–3°C) in KCl‐containing samples than in corresponding ones without KCl, suggesting that both SSB and SSB^H55Y^ tetramers are stabilized by chloride ions that inhibit intertetramer OB–CTP interactions and SSB condensation (Figure S3). Importantly, however, Glu showed a marked stabilizing effect (by about 20°C) on both SSB and SSB^H55Y^ tetramers, suggesting adaptations of SSB structure to the physiological intracellular anion (Figure S3).

**FIGURE 7:**
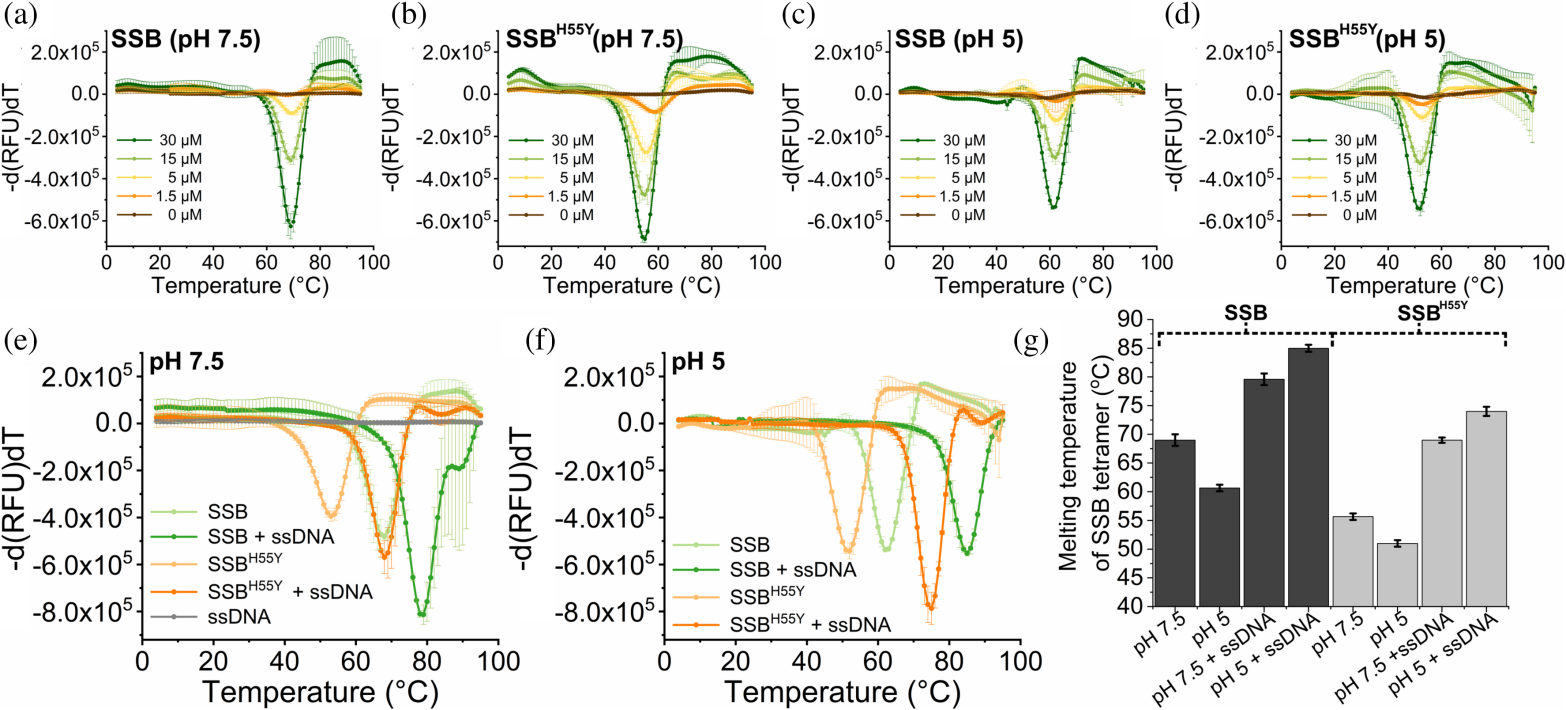
SSB^H55Y^ tetramers show reduced thermostability compared to wild‐type SSB. (a)–(d) ThermoFluor measurements of (a) and (c) SSB or (b) and (d) SSB^H55Y^ at different concentrations in buffers containing (a) and (b) 20 mM HEPES (pH 7.5) or (c) and (d), MES (pH 5), 5 mM Mg(OAc)_2_, and 50 mM KGlu. Means ± SD are shown for *n* = 3. (e), (f) ThermoFluor measurements of 15 μM SSB or SSB^H55Y^ in the presence and absence of 30 μM dT_79_ ssDNA in buffers containing (e) 20 mM HEPES (pH 7.5), 5 mM Mg(OAc)_2_ and 50 mM KGlu or (f) MES (pH 5), 5 mM Mg(OAc)_2_, and 50 mM KGlu. Means ± SD are shown for *n* = 3.(g) Melting temperature (*T*_m_) values (temperatures at peak *d*(RFU)/*dT* value) of SSB (dark gray) and SSB^H55Y^ (light gray) from panels (a)–(f) (Table S3). Means (*n* = 3) ± SD are shown. RFU, relative fluorescence units.

Subsequently, we compared the thermostability of both proteins across various protein concentrations (1.5–30 μM) under physiological (7.5) and acidic pH (5) conditions (Figure 7 and Table S3). While lowering the pH caused a relatively large (6–7°C) decrease in the *T*_m_ of SSB, this effect was markedly smaller for SSB^H55Y^ (2–3°C) (Figure 7). This result suggests that the positively charged form of H55 has an adverse impact on tetramer stability; notwithstanding the fact that SSB^H55Y^ is generally less stable than SSB, probably due to steric hindrance caused by the larger size of the tyrosine side chain compared to histidine.

We also examined the effect of ssDNA on SSB tetramer stability (Figure 7e–g). We found a large stabilizing effect of ssDNA upon SSB structure that persisted at different pH values (5 vs. 7.5) in both SSB and SSB^H55Y^, while the pH‐ and mutation‐dependent changes in *T*_m_ persisted also in the ssDNA‐bound form. Intriguingly, the stabilizing effect of ssDNA was for both SSB constructs greater at pH 5 (19–20°C) than at pH 7.5 (12–13°C), suggesting that ssDNA counteracts SSB intermonomer charge repulsions arising from amino acid side chain protonation (Figure 7e–g).

### 2.9 SSBdC condensates show reduced thermostability compared to SSB at physiological concentrations, while SSB^H55Y^ exhibits temperature‐induced aggregation

**FIGURE 8:**
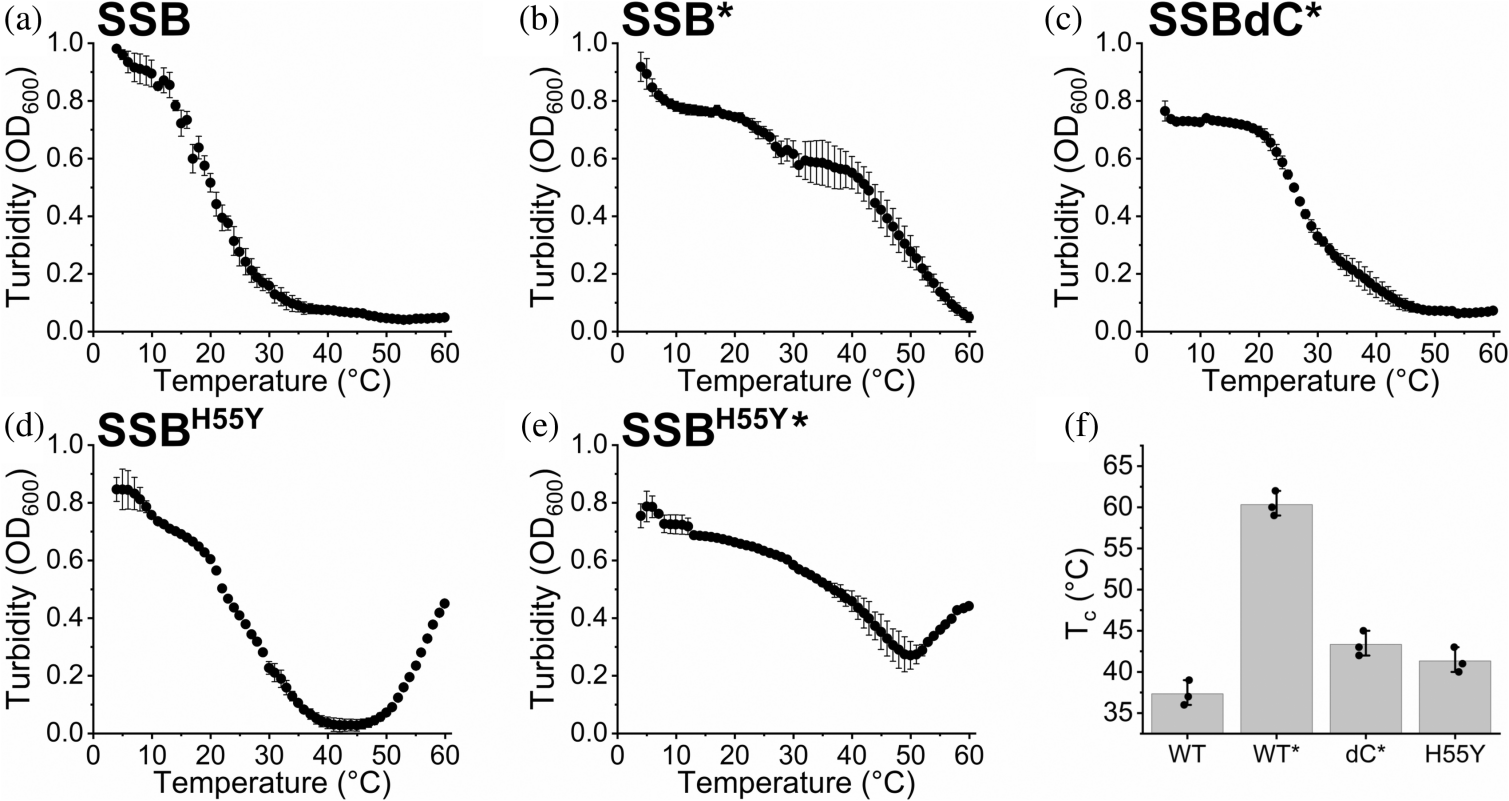
SSBdC condensates show reduced thermostability compared to wild‐type SSB, while SSB^H55Y^ exhibits temperature‐induced aggregation. (a)–(e) Temperature dependent turbidity (*OD*_600_) values of 5 μM SSB (approximate intracellular SSB concentration (Bobst et al., 1985)) in the absence (a) and presence (b) of 30 mg/mL PEG; 5 μM SSBdC in the presence of PEG (c); and 5 μM SSB^H55Y^ in the absence (d) and presence (e) of PEG. Samples were heated from 4 to 60°C at a rate of 0.2°C/min. Data points represent the mean ± SD of three independent experiments. * indicate experiments performed in the presence of PEG. (f) *T*_c_ values determined from experiments in panels (a)–(d). Columns show the mean ± SD, with individual experimental values shown as dots (Table S4). Columns labeled with * indicate experiments performed in the presence of PEG. *T*_c_ values were determined as turbidity values reaching buffer level. *T*_c_ values for SSB^H55Y^ in PEG could not be determined due to a temperature‐dependent increase in *OD*_600_ arising from protein precipitation/aggregation (panel e).

Besides the above experiments assessing tetramer thermostability, we also monitored the temperature dependence of condensation by the SSB, SSBdC, and SSB^H55Y^ constructs by performing temperature‐dependent turbidimetric (*OD*_600_) measurements (Figure 8 and Table S4). We performed these measurements at intermediate (quasi‐physiological) protein concentration (5 μM tetramers) in order to detect possible effects arising from varying tetramer stability. In the absence of PEG, both SSB and SSB^H55Y^ showed condensate disassembly around 37–42°C (Figure 8a, d, f). The increase in turbidity values above 45°C, which was seen only in the case of SSB^H55Y^, is indicative of protein precipitation and aggregation (Figure 8d, e). This finding is in line with the results of ThermoFluor measurements indicating protein unfolding and the disassembly of SSB^H55Y^ tetramers at comparable temperatures (Figures 7 and S3). The highly decreased thermostability of SSB^H55Y^ tetramers and the aggregation‐prone nature of the protein, both detected in the current study, provide an explanation for the heat sensitivity of the *ssb‐1 E. coli* strain (Meyer et al., 1980; Williams et al., 1984).

SSBdC was shown to be able to form condensates only in the presence of molecular crowders (Harami et al., 2020). In the present study, we found a greatly reduced thermostability for SSBdC condensates, compared to those of SSB, in the presence of PEG (Figure 8c, f).

## 3 DISCUSSION

The contribution of stereospecific interactions involving structured protein domains versus that of non‐stoichiometric interactions between intrinsically disordered segments to the condensation propensity of proteins is an aspect that needs to be elucidated both for better understanding of related physiological and pathological processes and for approaches involving future engineering and pharmacological targeting of protein condensation. Large‐scale modifications of SSB's IDL were previously shown to abolish the capability of the protein for condensation in the absence of molecular crowders (Kozlov et al., 2022). However, it remains unknown to what extent this effect is due to the loss of IDL–IDL interactions, or to significant alterations in the accessibility of the CTP for interactions with the OB folds of adjacent SSB tetramers. Our data show that CTP deletion renders SSB condensation dependent on molecular crowding, suggesting a significant but non‐essential role for the CTP in condensation, which can be brought about solely by the OB and (CTP‐less) IDL regions under these conditions (Figure 1). The CTP can either enhance condensation due to the direct interactions with the OB‐fold (Harami et al., 2020) or due to facilitation of the IDL–IDL interactions, by altering the IDL structure (Maffeo et al., 2022). Nevertheless, the contribution of OB–CTP interactions to SSB condensation propensity is further reflected in the markedly lower thermostability of SSBdC condensates compared to those formed by SSB (Figure 8).

Another remarkable feature of SSBdC discovered in this study is that its condensation is inhibited by glutamate, in stark contrast to that of SSB (Figure 2) (Harami et al., 2020; Kozlov et al., 2022). The condensation‐promoting effect of Glu on SSB was previously attributed to its unfavorable interactions with backbone amide (especially for Gly residues) and side chain amide groups of the IDL, promoting amide‐amide interactions between IDL regions (Kozlov et al., 2022). Our current findings showing that the presence of the CTP is essential to mediate Glu‐assisted condensation and that Glu decreases the CTP's affinity to the OB domain suggest that Glu might influence IDL structure in a CTP‐dependent way that brings about the earlier proposed effects on IDL amides. Moreover, we detected a massive positive effect of Glu on the thermostability of SSB tetramers, regardless of condensation state (Figure S3). Together with the large positive effect of the PEG molecular crowder on the thermostability of SSB condensates (Figure 8), these findings are indicative of adaptive fine‐tuning of SSB multimerization and condensation propensities to physicochemical conditions pertinent within bacterial cells (i.e., a glutamate‐rich, crowded environment). Nevertheless, we found that both SSB and SSBdC condensates were able to form over a broad pH range (about pH 4–9), despite the detected gradual weakening of the OB–CTP interaction with increasing pH (Figures 4, 5). Previous studies showed that SSB becomes dominantly monomeric below pH 4 (Shishmarev et al., 2014) but still remains folded. The lack of condensation below this pH further supports the role of SSB multivalency in condensation. In addition, the pH dependence of condensation in the 7–9 pH regime indicates that condensation, and presumably CTP binding, is mediated by protonated side chains. While further research is warranted, it is highly tempting to speculate that this broad pH profile may contribute to the extreme acid stress tolerance of *E. coli* or response to external pH fluctuations. Previously we proposed that ssDNA interferes with SSB condensation mainly by competing with OB–CTP interactions between adjacent tetramers (Harami et al., 2020). Unexpectedly, in the present study we found that the ssDNA dose‐dependent inhibition of SSB was retained in SSBdC (Figure 2c). This finding suggests that, although OB–CTP interactions do contribute to SSB condensation (Figures 1 and 8, see also above), the process is either also mediated via OB–IDL interactions between SSB tetramers, and/or that ssDNA binding to the OB fold influences condensation‐inducing IDL–IDL interactions. Further CTP‐independent contributions to ssDNA‐inhibited SSB condensation may come from previously proposed interactions of the IDL's PxxP motifs with the OB fold (Bianco, 2021). Furthermore, our finding that ssDNA exerts a markedly greater stabilizing effect on SSB tetramers at pH 5 than at pH 7.5 highlights the importance of ionic contributions to SSB‐ssDNA and/or SSB intratetramer OB–OB interactions (Figure 7).

SSB's physiological function as a protein–protein interaction hub has been proposed to be brought about in a large part by the interactions of the CTP with SSB's partner proteins (Bianco, 2021; Bonde et al., 2023; Shereda et al., 2008, 2009; Shinn et al., 2019; Su et al., 2014). Unexpectedly, in the present study, we found that the SSB binding partner RecQ helicase was significantly enriched in both SSB and SSBdC condensates (Figure 3). Moreover, these phenomena were also observed for RecQ variants harboring aa substitutions in RecQ's previously identified SSB‐binding interface that attenuated SSB binding in solution (Figure 3) (Harami et al., 2020). Previously we showed that the eGFP protein, unable to form specific interactions with SSB, failed to enrich inside SSB condensates even in the presence of PEG crowder, corroborating the proposition that stereospecific interactions are required to compensate for entropic losses incurred by inclusion of heteronomous protein species into condensates (Grigorev et al., 2023). Taking together, our new results suggests yet unidentified modes of interaction between SSB and RecQ, possibly involving the SSB IDL, which might also assist the inclusion of SSB's other partners into the condensates. Previously, the existence of an additional SSB interaction site outside of the RecQ WH domain was also proposed (Bagchi et al., 2018).

Multivalence of interactions between adjacent protein complexes has been identified as a definitive factor that brings about protein condensation (Banani et al., 2017). In the case of SSB, multivalence is chiefly brought about by homotetramerization. Therefore, to dissect such contributions to SSB condensation, we utilized the well‐characterized SSB^H55Y^ variant originally identified in the thermosensitive *ssb‐1* mutant *E. coli* strain (Meyer et al., 1980; Williams et al., 1984). *ssb‐1* cells also showed increased UV sensitivity (Carlini et al., 1993). SSB^H55Y^ was found to form unstable tetramers that dissociate into monomers as the SSB concentration was reduced from 10 to 0.5 μM (Bujalowski & Lohman, 1991a; Williams et al., 1984). This effect was attributed to disruption of the interface between SSB monomers by the aa substitution (Carlini et al., 1998). Our current study reveals both tetramerization and aggregation‐related factors that could contribute to the observed cellular phenotypes. We detected significantly reduced thermostability of SSB^H55Y^ tetramers compared to SSB (Figures 7 and S3). Interestingly, we found that the pH‐induced difference between tetramer dissociation temperatures between pH 5 (where H55 is expected to be mostly protonated) and pH 7.5 (where H55 is expected to be mostly deprotonated) was markedly greater for SSB than for SSB^H55Y^, suggesting that histidine protonation impairs the stability of SSB tetramers (Figure 7a–d). While SSB^H55Y^ showed WT‐like protein concentration and pH dependence of condensation (Figure 6), we detected thermally induced protein aggregation in the mutant protein, which is a likely contributor to the temperature‐dependent phenotype of *ssb‐1* cells. Another interesting finding was that Glu^−^, which is the major anion in bacteria, significantly increased the stability of SSB tetramers (both WT and mutant). This feature may be linked to the LLPS‐promoting effect of Glu^−^ (Kozlov et al., 2022) and reflect the adaptation of the SSB protein to the cellular environment in bacteria.

Taken together, our data show specific adaptations of the multimerization and condensation properties of the SSB protein to the conditions of its physiological functioning. We also show that, while IDL–IDL and/or OB–IDL interactions formed between SSBdC tetramers are sufficient to elicit condensation under special in vitro conditions, the glutamate‐inhibited nature of SSBdC condensation implies that this protein is unable to undergo condensation under cellular conditions. It remains to be determined in future in vivo SSB condensation engineering studies to what extent the loss of SSB's condensation and/or protein interaction capabilities contributes to the lethal phenotype of the SSBdC mutant (Antony et al., 2013; Waldman et al., 2016). All in all, our data demonstrate that the structural determinants of SSB condensation can be readily engineered in order to elucidate the physiological role of this feature in genome metabolism and to aid future efforts to target protein condensation for the development of new‐mechanism antibacterial agents.

## 4 MATERIALS AND METHODS

### 4.1 General reaction conditions

Condensation measurements were performed at 25°C in standard LLPS buffer containing 20 mM Tris‐Acetate (Tris‐OAc) pH 7.5, 50 mM KGlu, and 5 mM Mg(OAc)_2_, unless otherwise indicated. pH‐dependent condensation measurements were performed in buffers containing 20 mM citrate (pH range 3–4), 20 mM MES (pH range 4.5–6.5), or 20 mM Tris (pH range 7–10), 50 mM KGlu, and 5 mM Mg(OAc)_2_. For accurate pH determination, pH values were corrected based on controls using SSB storage buffer mixed with LLPS buffers in enlarged volumes, but preserving the volume proportions used in microscopic and turbidimetric studies.

### 4.2 Cloning, expression, purification, and fluorescent labeling of proteins

Cloning, expression, and purification of SSB, SSB^G26C^, SSBdC, and SSBdC^G26C^ were performed, as described in Harami et al. (2020). Briefly, plasmids encoding the SSB variants were transformed into *E. coli* ER2566 competent cells and were grown in 2YT medium at 37°C. Protein expression was induced using 1 mM isopropyl‐*β*‐D‐1‐thiogalactopyranoside at 18°C overnight. Cells were collected by centrifugation (3500*g*, 15 min). Pellets were resuspended in lysis buffer containing 50 mM Tris–HCl pH 8.3, 200 mM NaCl, 15 mM spermidine trihydrochloride, 10% sucrose, 1 mM EDTA, 1 mM PMSF, and 1 mM DTT. Cells were sonicated, and the cell debris was removed by centrifugation (19,000*g*, 50 min). Polimin P (10 (v/v) % stock, pH 7.0) was added to the supernatants at a final concentration of 0.4 (v/v) %. After 15 minutes of incubation, solutions were centrifuged (7000*g*, 20 min), and pellets were resuspended in a buffer containing 50 mM Tris–HCl pH 8.3, 400 mM NaCl, 1 mM EDTA, 1 mM DTT, and 20 (v/v) % glycerol. After 30 min of incubation, the remaining debris was removed by centrifugation (7000*g*, 20 min). Ammonium sulfate was added to the supernatants up to 27% saturation, causing SSB to reversibly precipitate. Precipitated fractions were collected by centrifugation (19,000*g*, 30 min). Pellets were resuspended in 50 mM Tris–HCl pH 8.3, 300 mM NaCl, 1 mM EDTA, 1 mM DTT, and 20 (v/v) % glycerol containing buffer, and then diluted in 50 mM Tris–HCl pH 8.3, 0.1 mM EDTA, 20 (v/v) % glycerol, and 1 mM DTT containing buffer to reach a final 50 mM NaCl concentration. Proteins were immediately applied to a Heparin column (HiTrap Heparin HP, GE Healthcare). Bound fractions were eluted applying a 50‐min linear salt gradient (50 mM–1 M NaCl). Eluted proteins were dialyzed to decrease the salt level to 100 mM NaCl and were further purified by applying them onto a MonoQ anion‐exchange column (5/50 GL Cytiva). The proteins were eluted with a salt gradient of 100 mM–1 M NaCl. Fractions containing SSB were dialyzed in storage buffer (50 mM Tris–HCl pH 7.5, 200 mM NaCl, 1 mM DTT, and 20 (v/v) % glycerol) and frozen in liquid nitrogen.

The plasmid coding for SSB^H55Y^ was generated from that coding for SSB‐WT by using the QuikChange mutagenesis kit (Agilent). Mutagenesis was verified by DNA sequencing. SSB^H55Y^ was purified as described for SSB‐WT.

Cloning, expression, and purification of RecQ‐WT, RecQ^R425A^, and RecQ^R499A^ were performed as described in Harami et al. (2020). Briefly, plasmids containing the RecQ coding sequences were transformed into *E. coli* ER2566 competent cells and were grown at 37°C in LB broth. Protein expression was induced using 0.2 mM IPTG at 18°C overnight. All purification steps were carried out at 4°C. Cells were collected as in the case of SSBs and were resuspended in a buffer containing 50 mM Tris–HCl pH 8.0, 500 mM NaCl, 1 mM EDTA, 10 (v/v) % glycerol, and 0.1 (v/v) % Triton X‐100. After sonication, the cell debris was removed by centrifugation (36,000*g*, 30 min), and the supernatant was applied to a chitin column (Chitin resin, NEB). After washing, the column was treated with buffer containing 50 mM DTT overnight. Eluted proteins were applied to a heparin column equilibrated with a buffer comprising 50 mM Tris–HCl pH 7.5, 200 mM NaCl, 0.1 mM EDTA, and 1 mM DTT. The bound proteins were eluted by applying a 50‐minute linear salt gradient (200 mM–1 M NaCl). Protein fractions containing RecQ were collected and dialyzed in storage buffer (50 mM Tris–HCl pH 7.5, 200 mM NaCl, 0.1 mM EDTA, 1 mM DTT, 10 (v/v) % glycerol).

Purity was verified by SDS‐PAGE for all proteins. Concentrations of purified proteins were determined using the Bradford method. Purified proteins were flash‐frozen and stored in liquid N_2_ in 20‐μL droplets.

SSB variant harboring the G26C aa substitution was labeled with FITC (Fluorescein 5‐isothiocyanate, Sigma‐Aldrich) or Alexa555 (Alexa Fluor 555 C2 Maleimide, Thermo Fisher) as in Harami et al. (2020). RecQ variants were labeled on their N‐terminus with Alexa488 (Alexa Fluor 488 carboxylic acid, succinimidyl ester, Thermo Fisher) as in Harami et al. (2020).

### 4.3 Turbidity measurements

Turbidity (*OD*) of 40‐μL samples was measured at 600 nm at 25°C in transparent 384‐well plates (Nunc Thermo Fisher PN:242757) in a Tecan Infinite Nano plate reader instrument. Measurements were carried out in LLPS buffers (see above). Condensation reactions were initiated by mixing aliquots of non‐condensed SSB samples with solutions containing the specified effectors. The optical path length for all experiments was standardized at 0.476 cm. SSB in a non‐condensed state and other buffer components showed no significant light absorption at 600 nm (*OD*_600_ < 0.05), as confirmed by absorption spectra. When condensation occurred, apparent light absorption was detected due to light scattering of condensed droplets and/or aggregates.

### 4.4 Fluorescence polarization assays for CTP and ssDNA binding

In CTP binding assays, 15 nM fluorescein‐labeled SSB CTP (flu‐CTP, Flu‐MDFDDDIPF) was titrated with SSB or SSBdC in LLPS buffer complemented with 30 mg/mL PEG, 20 μM dT_79_, 300 mM NaCl, 300 mM KGlu, or was set to different pH values as described above in General reaction conditions. In ssDNA binding assays, 10 nM of fluorescein‐labeled ssDNA (ATTTTTGCGGATGGCTTAGAGCTTAATTGCGCAACG‐Flu) was titrated with increasing concentrations of SSB, SSBdC, or SSB^H55Y^ in LLPS buffers.

Fluorescence polarization of 20‐μL samples was measured in 384‐well low‐volume nontransparent microplates (Greiner Bio‐one, PN:784900) using a Synergy H4 Hybrid Multi‐Mode Microplate Reader (BioTek) at 25°C. Three independent experiments were carried out. *K*_d_ values were determined by using a quadratic binding equation (Wang, 1995) using OriginLab 8.0 software (Microcal Corp.).

### 4.5 ThermoFluor assays

ThermoFluor measurements of 40‐μL samples were carried out in a StepOnePlus Real Time PCR instrument by mixing indicated concentrations of unlabeled SSB proteins with Sypro Orange dye in buffers containing 20 mM HEPES (pH 7.5) or MES (pH 5), 5 mM Mg(OAc)_2_, and 50 mM KGlu if other is not indicated. In this method, the fluorescent dye binds to exposed hydrophobic protein regions upon thermally induced unfolding, and a consequent elevation in fluorescence is recorded.

### 4.6 Temperature profiles of condensate formation

Solutions containing SSB variants at 5 μM concentration (the approximate intracellular SSB concentration (Bobst et al., 1985)) were mixed at 4°C, pipetted into cuvettes with a 1‐cm path length, and inserted into a Cary E4 spectrophotometer with an attached water thermostat. Cuvettes were sealed to prevent evaporation. Samples were heated to 60°C at a rate of 0.2°C/min, with concomitant measurement of *OD*_600_ in 1°C intervals. Thermal Software (version 3.0) was used for the measurements. Experiments were carried out in triplicate.

### 4.7 Fluorescence microscopy

A Nikon Eclipse Ti‐E TIRF microscope was used with an apo TIRF 100× oil immersion objective (numerical aperture (NA) 1.49) in epifluorescence mode in SSB condensation experiments. For fluorescence excitation, a 543‐nm laser (25‐LGP‐193–230, Melles Griot) and a Cyan 488‐nm laser (Coherent) were used depending on the excitation spectra of the applied fluorescent probes. Fluorescence was directed to a ZT405/488/561/640rpc BS dichroic mirror and captured using a Zyla sCMOS (ANDOR) camera. Sample chamber surfaces were blocked using 1 mg/mL Blocking Reagent (Roche) before measurements, unless otherwise indicated. Blocking reagent was applied for 20 min in buffers used for measurements and then washed out using the reaction buffer. 20‐μL SSB samples were imaged in μ‐Slide Angiogenesis (Ibidi) microscope slides at 25°C, and the condensate formation was followed. Images were captured using NIS‐Elements AR (Advanced Research) 4.50.00 software, employing 2 × 2 pixel binning and a 200‐ms laser exposure time during acquisition. Images were background corrected unless otherwise indicated.

### 4.8 Image processing for fluorescence microscopy

Fiji (ImageJ) software was used for analysis of raw microscopic images. During analysis of images monitoring SSB condensation, image stacks and montages were generated from experimental sets to uniformly adjust brightness and contrast utilizing Fiji's automatic detection algorithm. For representative purposes, the built‐in rolling ball background correction algorithm was used for background correction, unless otherwise specified. Middle image sections were used to quantify the extent of condensation, mean gray values, and total droplet areas as follows. A stack of three background‐uncorrected images was created from three distinct fields of view recorded for a given condition. A square region (600 × 600 pixels) centered on each image was chosen for further examination (referred to as the middle region of interest (ROI)) to exclude effects arising from uneven illumination at the periphery of the original images. Stack Fitter plugin was used to calculate mean gray value (computed as the sum of pixel intensities divided by the total pixel number) for each ROI. Average mean gray values were then calculated based on three analyzed images. The built‐in thresholding algorithm was used to assess the total droplet area in the same regions by establishing a signal threshold to distinguish between the background and the signal arising from the condensates. Fiji's built‐in particle analyzer algorithm (with the smallest detected size set to 0.2 μm^2^ and circularity parameter set to 0.1–1) was utilized to outline and identify the area of the fluorescent condensates. Determined areas were combined for each image, and results were averaged over the three analyzed regions. Enrichment of partner proteins inside condensates was calculated as described in Harami et al. (2020).

## AUTHOR CONTRIBUTIONS

**Zoltán J. Kovács:** Conceptualization; data curation; formal analysis; investigation; methodology; validation; visualization; writing – original draft; writing – review and editing. **Péter Ecsédi:** Conceptualization; methodology; data curation; investigation; validation; formal analysis; visualization; writing – original draft; writing – review and editing. **Gábor M. Harami:** Methodology; formal analysis; writing – review and editing. **János Pálinkás:** Conceptualization; methodology. **Mina Botros:** Investigation; validation; methodology; visualization. **Lamiya Mahmudova:** Investigation; validation; methodology. **Viktoria Katran:** Investigation; writing – review and editing. **Dávid Érfalvy:** Data curation; validation; investigation. **Miklós Cervenak:** Investigation; methodology. **László Smeller:** Resources; methodology. **Mihály Kovács:** Conceptualization; funding acquisition; project administration; resources; supervision; writing – review and editing.

## CONFLICT OF INTEREST STATEMENT

The authors have no conflicts of interest to declare.

## Supporting information


**Data S1.** Supporting Information.

## Data Availability

The data that support the findings of this study are available from the corresponding author upon reasonable request.
