# Supplementary material for: Fine‐tuned interactions between globular and disordered regions of single‐stranded DNA binding (SSB) protein are required for dynamic condensation under physiological conditions
Source: Protein Sci. 2025 Mar 27;34(4):e70109. doi: 10.1002/pro.70109 (PMC11947617; doi:10.1002/pro.70109)
Supplement: Supplementary file 1 — Data S1. Supporting Information. [file PRO-34-e70109-s001.docx]

**SUPPLEMENTARY MATERIAL**

**
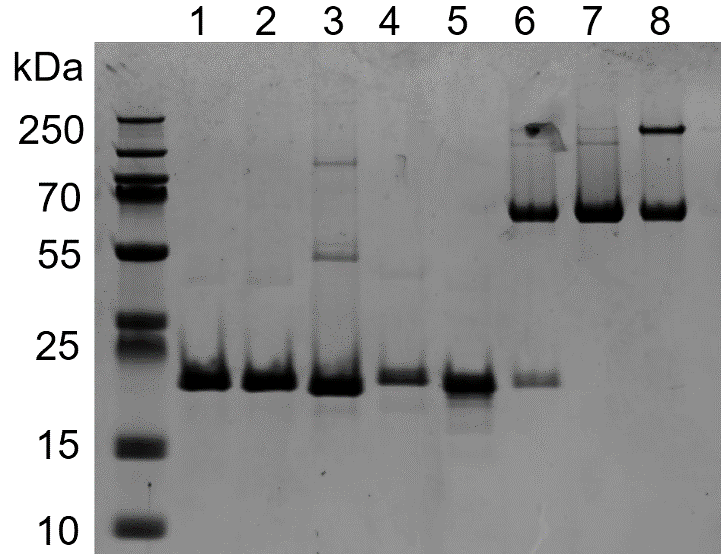
**

**Fig. S1: SDS polyacrylamide gel electrophoresis of protein constructs used in this study**

Shown is Coomassie Brilliant Blue R 250-stained SDS-PAGE of purified SSB (1), SSB^H55Y^ (2), SSBdC (3), Alexa555-labeled SSB (4), Alexa555-labeled SSBdC (5), Alexa488-labeled WT RecQ (6), Alexa488-labeled RecQ^R425A^ (7), and Alexa488-labeled RecQ^R499A^ (8) protein constructs. 5 μg protein was loaded in each lane. 4–20 % precast SDS polyacrylamide gel (BIO-RAD #4561095) and PageRuler Plus Prestained Protein Ladder were used in the experiment.


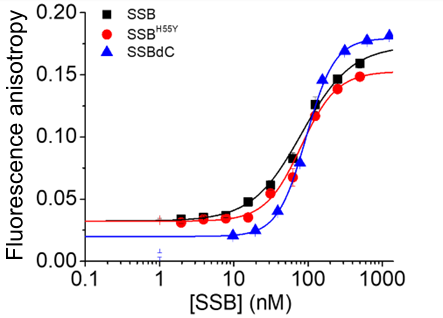


**Fig. S2: ssDNA binding by SSB is not impaired by either the deletion of the C-terminal peptide or the H55Y amino acid substitution**

Fluorescence anisotropy titrations of 10 nM 3’-fluorescein labeled, 36-mer ssDNA (see Materials and Methods) with increasing concentrations of SSB, SSBdC and SSB^H55Y^ constructs. Solid lines show best fits based on the Hill equation. Best-fit parameters were as follows: SSB, *K*_d_ = 82 ± 12 nM, *n* (Hill coefficient) = 1.3 ± 0.1; SSBdC, *K*_d_ = 94 ± 7 nM, *n* = 2.3 ± 0.3; SSB^H55Y^, *K*_d_ = 81 ± 8 nM, *n* = 1.8 ± 0.2. Means ± SEM are shown for *n* = 3.


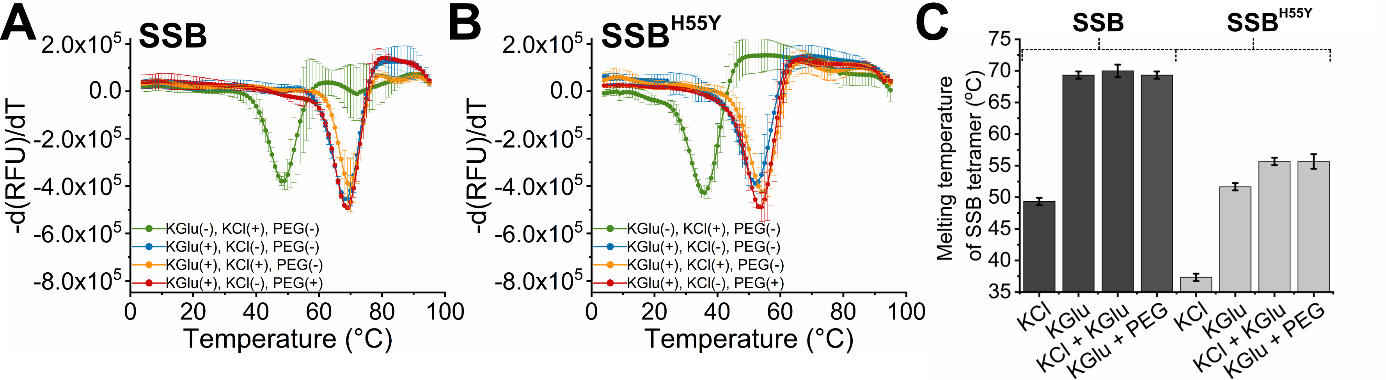


**Fig. S3: ThermoFluor assay shows reduced tetramer stability of SSB^H55Y^ compared to SSB, and a marked tetramer-stabilizing effect of glutamate for both constructs**

**(A-B)** ThermoFluor measurements performed on 15 µM **(A)** SSB and **(B)** SSB^H55Y^ in a buffer containing 20 mM HEPES (pH 7.5) and 5 mM Mg(OAc)_2_, with or without 200 mM KCl, 50 mM KGlu, and 30 mg/ml PEG. Data points show means ± SD of three independent experiments.

**(C)** Melting temperatures (*T*_m_, temperature at peak d(RFU)/dT) of SSB (dark grey) and SSB^H55Y^ (light grey) from panels **A-B (Table S3)**. Means ± SD are shown for *n* = 3. RFU, relative fluorescence units.

|  | **KCl (mM)** | **NaCl (mM)** | **KGlu (mM)** | **NaGlu (mM)** | **KOAc (mM)** | **NaOAc (mM)** |
| --- | --- | --- | --- | --- | --- | --- |
| SSB with BSA | 91 ± 1 | 110 ± 10 | > 500 | > 500 | > 500 | > 500 |
| SSBdC with BSA | 40 ± 2 | 37 ± 4 | 66 ± 2 | 70 ± 5 | 120 ± 10 | 100 ± 10 |
| SSB with PEG | 110 ± 2 | 90 ± 6 | > 500 | > 500 | 250 ± 180 | 290 ± 200 |
| SSBdC with PEG | 41 ± 1 | 32 ± 5 | 73 ± 19 | 60 ± 15 | 55 ± 18 | 48 ± 17 |

**Table S1. Half-maximal effective salt concentrations to disrupt EcSSB condensates**

Values were determined in turbidity assays (**Fig. 2**) and show the mean ± SD of the best-fit parameters (based on the Hill equation) of three independent measurements.

**Table S2. *K*_d_ values of binding of labeled C-terminal peptide (flu-CTP) to SSB constructs**

| \|  \| ***K*_d_ (µM)** \| \| --- \| --- \| \| SSB \| > 100 \| \| SSBdC \| 2.4 ± 1.0 \| \| SSB with PEG \| > 100 \| \| SSBdC with PEG \| 0.08 ± 0.02 \| \| SSB with 300 mM KCl \| > 100 \| \| SSBdC with 300 mM KCl \| > 100 \| \| SSBdC with 20µM ssDNA \| > 100 \| \| SSBdC with 300 mM KGlu \| 12 ± 3 \| \| SSBdC with 300 mM KGlu and PEG \| 5.9 ± 1.0 \| \| SSBdC at pH 3 \| 0.33 ± 0.11 \| \| SSBdC at pH 3.5 \| 0.60 ± 0.15 \| \| SSBdC at pH 4 \| 0.93 ± 0.15 \| \| SSBdC at pH 4.5 \| 0.51 ± 0.39 \| \| SSBdC at pH 5 \| 0.65 ± 0.32 \| \| SSBdC at pH 5.5 \| 1.2 ± 0.1 \| \| SSBdC at pH 6 \| 0.85 ± 0.34 \| \| SSBdC at pH 6.5 \| 1.0 ± 0.5 \| \| SSBdC at pH 7 \| 0.94 ± 0.21 \| \| SSBdC at pH 7.5 \| 2.0 ± 0.3 \| \| SSBdC at pH 8 \| 2.2 ± 0.5 \| \| SSBdC at pH 8.5 \| 2.6 ± 0.4 \| \| SSBdC at pH 9 \| 5.0 ± 0.2 \| \| SSBdC at pH 9.5 \| 6.5 ± 0.5 \| \| SSBdC at pH 10 \| 6.9 ± 1.2 \| |
| --- | --- | --- | --- | --- | --- | --- | --- | --- | --- | --- | --- | --- | --- | --- | --- | --- | --- | --- | --- | --- | --- | --- | --- | --- | --- | --- | --- | --- | --- | --- | --- | --- | --- | --- | --- | --- | --- | --- | --- | --- | --- | --- | --- | --- | --- | --- | --- | --- | --- | --- |
| Dissociation constants (*K*_d_) were determined in FP assays (**Fig. 5**). Values reported are means ± SD of best-fit parameters of three independent measurements. Fits were based on a quadratic binding equation. |

**Table S3. *Tm* values of SSB constructs from Fig. 7 and Fig. S3**

|  | **Tm (^o^C)** |
| --- | --- |
| SSB pH 7.5 | 69.0 ± 1.0 |
| SSB pH 5 | 60.6 ± 0.6 |
| SSB pH 7.5 + ssDNA | 79.6 ± 1.1 |
| SSB pH 5 + ssDNA | 84.8 ± 0.3 |
| SSB^H55Y^ pH 7.5 | 55.4 ± 0.4 |
| SSB^H55Y^ pH 5 | 51.0 ± 0.6 |
| SSB^H55Y^ pH 7.5 + ssDNA | 69.0 ± 0.4 |
| SSB^H55Y^ pH 5 + ssDNA | 74.0 ± 0.9 |
| SSB (KCl) | 49.3 ± 0.5 |
| SSB (KGlu) | 69.2 ± 0.6 |
| SSB (KCl +KGlu) | 70.1 ± 1.4 |
| SSB (KGlu + PEG) | 69.3 ± 0.6 |
| SSB^H55Y^ (KCl) | 37.3 ± 0.6 |
| SSB^H55Y^ (KGlu) | 51.6 ± 0.5 |
| SSB^H55Y^ (KCl +KGlu) | 55.5 ± 0.6 |
| SSB^H55Y^ (KGlu + PEG) | 55.1 ± 0.4 |
|  |  |

Values were determined in ThermoFluor assays (Fig. 7 and S3) and show the means ± SD (*n* = 3).

**Table S4. *Tc* values of SSBs from Fig. 8**

|  | **Tc (^o^C)** |
| --- | --- |
| WT | 37.3 ± 1.5 |
| WT* | 60.4 ± 1.4 |
| dC* | 43.3 ± 1.0 |
| H55Y | 41.0 ± 1.2 |
|  |  |

Values were determined in temperature dependent turbidity assays (Fig. 8) and show the means ± SD (*n* = 3). * indicates presence of PEG.
